# Spot the Difference: Morphometrics Reveals Key Traits for Distinguishing Two Cryptic Juvenile Skate

**DOI:** 10.1002/ece3.74305

**Published:** 2026-09-04

**Authors:** Sophie Loca, Sara Fullerton, Caroline Bradley, James Thorburn, Amy Garbett, Jack Burton, Amber Keenan, Daniel Wise, Lauren Smith, Chris Rickard, Thomas Barreau, Samuel Smith, Paulo A. Prodöhl, Patrick C. Collins

**Affiliations:** ^1^ School of Biological Science Queen's University Belfast Belfast UK; ^2^ Centre for Conservation and Restoration Science, School of Applied Sciences Edinburgh Napier University Edinburgh UK; ^3^ Orkney Skate Trust Stromness Orkney UK; ^4^ Saltwater Life Aberdeenshire UK; ^5^ Shark and Skate Scotland Aberdeenshire UK; ^6^ Muséum National d'Histoire Naturelle Station Marine de Dinard Dinard France

**Keywords:** bycatch, common skate, conservation, critically endangered, *Dipturus batis*, *Dipturus intermedius*

## Abstract

Accurate species identification is essential for effective management of threatened species, especially those with overlapping distributions and similar morphologies. Skate (Order *Rajiformes*) exhibit a highly conserved superficial morphology, which has resulted in a long history of taxonomic confusion and misidentification within this group. The flapper skate (*Dipturus intermedius*) and common blue skate ( *D. batis* ) are prime examples of this challenge; both species inhabit the Northeast Atlantic and share a complicated, intertwined taxonomic history. Given their overlapping distributions, precise species identification is necessary to ensure targeted management actions. Despite the availability of reliable morphological keys for larger specimens, identification of juveniles remains challenging because many diagnostic features are underdeveloped or obscured by damage. This study aimed to identify additional traits for differentiating juvenile (< 100 cm) flapper and common blue skate by employing morphological approaches validated through molecular techniques. The analysis focused on identifying distinct features that remain detectable in damaged specimens, ensuring the method's practical applicability in field conditions. We identified four robust features that can be used to differentiate juveniles of the two species: ventral coloration, disc shape, anterior disc margin shape and rostrum shape. The findings presented here enhance data accuracy for juvenile flapper and common blue skates, support targeted conservation actions, and contribute to the effective management and long‐term recovery of these threatened species.

## Graphical abstract

Juvenile flapper (*Dipturus intermedius*) and common blue ( *D. batis* ) skates are difficult to distinguish due to overlapping distributions and similar morphologies, which hinders monitoring efforts. Using geometric morphometrics and image analysis, this study identified four robust features thay reliably differentiate juveniles: ventral coloration, disc shape, anterior disc margin shape and rostrum shape. These traits provide practical tools for accurate species identification, supporting conservation, management and long‐term recovery of these threatened skates.

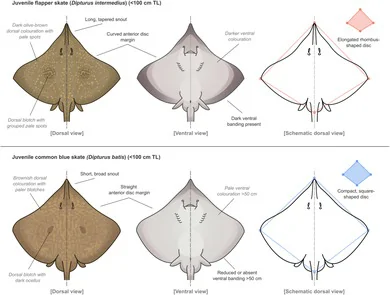

## 1 Introduction

Accurate species identification is essential for the effective management of threatened species, particularly those with overlapping distributions and similar morphological characteristics (Siskey et al. 2019). Skate (Order *Rajiformes*) exhibit a highly conserved superficial morphology, which has led to a long history of taxonomic confusion and misidentification within the group (e.g., Last et al. 2008; Iglésias et al. 2010; Geraci et al. 2017; Siskey et al. 2019; Carugati et al. 2022). Historically, the issue was exacerbated by the common practise of landing skates and rays under a single label (‘Skates and rays’; Ellis et al. 2010; Siskey et al. 2019; Dulvy et al. 2000), reflecting both their limited commercial value and masking species‐specific declines in abundance (Brander 1981; Iglésias et al. 2010; Siskey et al. 2019; Dulvy et al. 2000). While reporting practises have improved in recent years (Ellis et al. 2010; Silva et al. 2012; Simpson and Sims 2016), species misidentification continues to be significant issue affecting the accuracy of contemporary landings data (Iglésias et al. 2010; Siskey et al. 2019; Garbett et al. 2023). These data quality issues hinder conservation efforts, as reliable species‐specific information is essential for developing and implementing effective management strategies (Siskey et al. 2019; Loca, Garbett, et al. 2025). Given the precarious conservation status of many skate species (e.g., Dulvy et al. 2021), establishing standardised and reliable methods of species identification are of critical importance.

The flapper skate, *Dipturus intermedius* (Parnell, 1837), and common blue skate, *D. batis* (Linnaeus 1758), exemplify this challenge, as both species inhabit the Northeast Atlantic and share a complicated and intertwined taxonomic history (Bache‐Jeffreys et al. 2021; Garbett et al. 2023). Intense fishing pressure throughout the 20th century led to significant declines and local extirpations, resulting in their current Critically Endangered status (Brander 1981; Walker and Hislop 1998; Ellis, McCully‐Philips, et al. 2024; Loca, Collins, et al. 2025). Due to their superficial morphological similarity, the flapper skate and common blue skate were mistakenly considered a single species (the common skate, then *D. batis* (Linnaeus 1758)) until around a decade ago, when two independent studies revealed two distinct clades within the ‘common skate’ species (Griffiths et al. 2010; Iglésias et al. 2010). The proposed species classifications were formally recognised in 2021 (Ellis, McCully‐Philips, et al. 2024).

Both the flapper skate and common blue skate exhibit slow (k‐selected) life histories, producing relatively few offspring that require up to two decades to reach reproductive maturity (Brander 1981; Iglésias et al. 2010; Thorburn et al. 2023), resulting in exceptionally low population growth rates (Hutchings et al. 2012; Régnier et al. 2021). Consequently, successful juvenile recruitment is an important component of population persistence, as individuals that survive to maturity may contribute to reproduction over many decades (Ward‐Paige et al. 2012; Kindsvater et al. 2016; Mejía et al. 2025). To support vulnerable early life stages, many large skate species rely on critical habitats, such as egg nurseries, which are integral to enhancing survival rates (Heupel et al. 2007; Martins et al. 2018; Phillips et al. 2021; Dodd et al. 2022). These habitats make excellent conservation targets as they can provide disproportionate benefits for population recovery (U.S. Public Law 1996). However, juvenile flapper and common blue skate, along with their critical habitats, are currently subject to significant fishing pressure throughout their distribution (Garbett et al. 2023; Ellis, Gordon, et al. 2024; Loca, Collins, et al. 2025). This exposure underscores the need to understand and monitor juvenile recruitment and survival as part of effective species‐specific management strategies (Garbett et al. 2021).

Given their overlapping distributions (Frost et al. 2020; Bache‐Jeffreys et al. 2021), and distinct ecologies (Iglésias et al. 2010; Frost et al. 2020), accurate species identification is critical to ensure that management actions are appropriately targeted (Garbett et al. 2021; Loca, Garbett, et al. 2025). Although effective molecular techniques have been developed to distinguish these species (Griffiths et al. 2010; Bache‐Jeffreys et al. 2021; Carugati et al. 2022; Garbett et al. 2023), their application requires specialised equipment, time and expertise, which can limit their use in routine monitoring. In contrast, morphological methods offer a more accessible approach for species identification that can be utilised by personnel with minimal training and can be applied to historical datasets containing morphometric data (Garbett et al. 2023). Iglésias et al. (2010) identified five key traits for differentiating the flapper skate from the common blue skate: eye colour, wing spot patterns, lateral tail thorns, dorsal fin interspaces and dentition. However, the consistent identification of juveniles remains challenging, as some of these traits are less developed in smaller individuals or may be obscured by capture‐related epidermal trauma. Consequently, the misidentification of juveniles is a persistent issue, reducing the utility of much of the existing fisheries management data (e.g., DATRAS) for informing conservation efforts (Garbett et al. 2021; Ellis, McCully‐Philips, et al. 2024).

This paper addresses the need for improved identification methods for juvenile flapper and common blue skates. By integrating morphological and molecular approaches, we aim to develop a validated framework for distinguishing these two species in the field. Morphological analyses will focus on key distinguishing features that remain identifiable even when the specimens are damaged, while molecular data will provide independent confirmation of species identity and validation of the morphological framework.

## 2 Materials and Methods

### 2.1 Sample Acquisition

During the 2021 Marine Institute Irish Groundfish survey, 94 fin clips were collected from deceased *Dipturus* skate species, and stored in 2 mL tubes containing 99% molecular‐grade ethanol for subsequent genetic analysis. Photographs of both the dorsal and ventral surfaces were taken for each individual, capturing the entire body from the tip of the nose to the end of the tail. A ruler was included in each image to provide a consistent scale in mm. Individuals measuring less than 100 cm total length were retained for further analysis (*n* = 84), ensuring that only juvenile individuals of both species were included while encompassing both early‐ and mid‐juvenile stages (Iglésias et al. 2010; Ellis, Gordon, et al. 2024).

### 2.2 Ethics Statement

All specimens used in this study were obtained as deceased fisheries bycatch. As the research involved analysis of previously deceased specimens only, with no live animal handling or experimental manipulation, ethical approval was not required.

### 2.3 COI Barcoding

Genomic DNA was extracted from tissue samples using Wizard SV 96 Genomic Purification System (Promega) following the manufacturer's protocol. DNA quality was assessed by comparing it against *Hind*III digested ʎ DNA on 0.8% 0.5X TBE GelRed‐stained agarose gels. Mitochondrial (mtDNA) variation was analysed by directly sequencing of PCR amplified products (668 bp) of the COI mitochondrial region using the primer set DiptCOI (DiptCOIF—GTCGGAACTGGCCTAAGTCT and DiptCOIR—TGCCGGGTCAAAGAAAGTTG; Bradley et al., submitted). These primers were designed based on *Dipturus* species consensus sequences available on GenBank. PCR amplifications were conducted in a 50 μL reaction volume containing 0.4 units of Invitrogen *Taq* DNA polymerase, 1X Invitrogen *Taq* polymerase buffer, 1.5 mM MgCl_2_, 0.2 mM dNTPs, 10 pM of each primer and 100 ng of genomic template DNA. The PCR protocol consisted of an initial denaturation at 95°C for 15 min, followed by 30 cycles of denaturation at 95°C for 1 min, primer annealing at 55°C for 1 min and extension at 72°C for 2 min 30 s. A final extension step at 72°C for 10 min was included. PCR products were purified using the Invitrogen Purelink Quick PCR Purification Kit and sequenced by Eurofins Genomics using a 3730XL Automatic DNA Analyser.

DNA sequence chromatograms were manually reviewed and edited using Geneious Prime (Biomatters Ltd., Kearse et al. 2012). DNA sequence alignments were performed using the CLUSTAL Omega multiple alignment algorithm within the Geneious Prime sequence alignment editor. Individuals were reliably identified to species based on 12 diagnostic sites within this particular mtDNA region, which distinguish between flapper skate and blue skate (Bradley et al., submitted). To illustrate the genetic differentiation between species, a phylogenetic tree was reconstructed in Geneious Prime using the PhyML plugin (Guindon et al. 2010) under the Kimura two‐parameter (K80) substitution model with 1000 bootstrap replicates. The resulting tree is presented in the Appendix (Figure S1).

### 2.4 Morphometric Analyses

To explore morphological features that could be used to distinguish the two skate species, three separate landmark‐based geometric morphometric analyses were conducted according to the protocol given by (Zelditch et al. 2012; Franklin et al. 2014; Martinez et al. 2016; López‐Romero et al. 2025). Due to variation in image quality, each analysis employed a subset of the full image dataset. Dorsal images were chosen to ensure adequate lighting, focus and coverage of the required feature. A total of 37 images were chosen for the full disc (FD) analysis (18 × blue skate, 19 × flapper skate), 26 for the anterior disc margin (ADM) analysis (11 × blue skate, 15 × flapper skate) and 25 for the head and rostrum (HR) analysis (13 × blue skate, 12 × flapper skate; Table S1). Each image was rotated and cropped to capture the full disc, excluding the tail.

**FIGURE 1:**
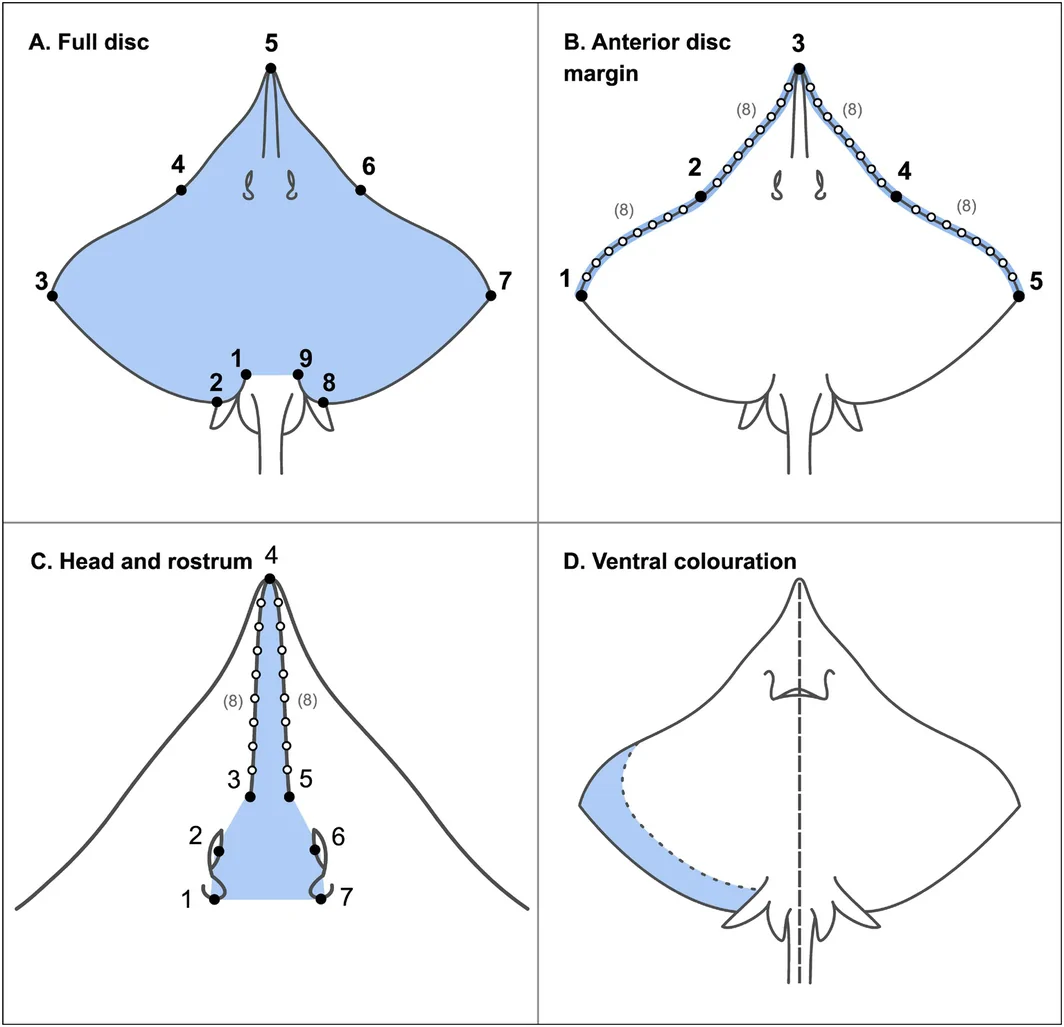
Features examined in a morphological study of juvenile common blue skate ( *D. batis* ) and flapper skate ( *D. intermedius* ). A. Full disc (FD) morphometric analysis showing 9 homologous landmarks (1. Left pectoral fin insertion, 2. Left posterior disc apex, 3. Left disc corner, 4. Left concave midpoint of anterior disc margin, 5. Tip of snout, 6. Right concave midpoint of anterior disc margin, 7. Right disc corner, 8. Right posterior disc apex, 9. Right pectoral fin insertion), B. Anterior disc margin (ADM) morphometric analysis showing 5 homologous landmarks and 32 non‐homologous landmarks (1. Left disc corner, 2. Left concave midpoint of anterior disc margin, 3. Tip of snout, 4. Right concave midpoint of anterior disc margin, 5. Right disc corner), C. Head and rostrum (HR) morphometric analysis showing 7 homologous landmarks and 16 non‐homologous landmarks (1. Left anterior margin of spiracle, 2. Left orbit centre, 3. Left base of rostrum, 4. Tip of snout, 5. Right base of rostrum, 6. Right orbit centre, 7. Right anterior margin of spiracle), D. Ventral colouration image analysis. Homologous landmarks and non‐homologous semi‐landmarks are represented by black circles and white circles, respectively. Blue shaded regions highlight the region of interest for each analysis.

Images were organised into separate folders and randomised digital reference (TPS) files were generated for each using *tpsUtil* (v1.83; Rohlf 2023), prior to landmark digitisation in *tpsDig2* (v2.31; Rohlf 2017). Homologous landmarks corresponded to anatomically distinct points that could be reliably identified, while semi‐landmarks were used to capture curvature along the outlines between homologous landmarks (Figure 1). Curves were defined using the trace function in *tpsDig2*, with a fixed number of equidistant points along each curve. These curves were then converted to semi‐landmarks and appended to the homologous landmark dataset using *tpsUtil*. Size differences between samples were standardised by setting the scale in *tpsDig2* with the measure tool, based on the scale bar present in the image. Following digitisation, a total of nine homologous landmarks were defined for the full disc (FD; Figure 1A), five homologous and 32 semi‐landmarks for the anterior disc margin (ADM; Figure 1B), and seven homologous and 16 semi‐landmarks for the head and rostrum (HR; Figure 1C). The TPS files were exported for further analyses.

Geometric morphometric analyses were performed in RStudio (Posit team 2026) using functions from the *geomorph* package unless otherwise stated (Baken et al. 2021; Adams et al. 2026). TPS files were imported using the ‘readland.tps’ function, and coordinates aligned using Generalised Procrustes Analyses (‘gpagen’ function), which removed non‐shape variation due to sample size, orientation and position (Klingenberg 2011; Zelditch et al. 2012). Differences in mean shape between species were assessed using Procrustes ANOVA (‘procD.lm’ function) with 1000 permutations of Procrustes‐aligned coordinates. Although Generalised Procrustes Analysis removes isometric differences in size, shape variation associated with allometry may remain. Centroid size is defined as the square root of the sum of squared distances between landmarks, and the shape centroid can be used to investigate allometry in morphometric research (Frédérich and Vandewalle 2011). Therefore, the effects of log‐transformed centroid size and sex on shape variation were assessed using Procrustes ANOVA.

A principal component analysis (PCA) was performed on Procrustes coordinates to summarise major axes of shape variation using the ‘gm.prcomp’ function. Species differences in shape were then evaluated using linear discriminant analysis (LDA; ‘lda’ function from the *MASS* package; Venables and Ripley 2002), equivalent to canonical variate analysis (CVA). CVA identifies the linear combination of variables that maximises separation among predefined groups while minimising within‐group variation (Campbell and Atchley 1981; Klingenberg and Monteiro 2005). To reduce dimensionality and limit overfitting associated with high‐dimensional landmark data and a relatively small sample size, the analysis was performed on principal component (PC) scores rather than Procrustes coordinates (Mitteroecker and Schaefer 2022). Principal components explaining at least 90% of cumulative shape variation were retained for each dataset (FD: PC1–PC8; ADM: PC1–PC5; HR: PC1–PC6). The contribution of each retained PC to the first canonical variate (CV1) was quantified from the LDA coefficients, and each specimen was assigned a CV1 score. The distribution of CV1 scores for each species was visualised using kernel density plots generated with the *ggplot2* package (v3.5.2; Wickham 2016). Species classification performance for each trait was evaluated using leave‐one‐out cross‐validation of the LDA model. Prediction accuracy, Cohen's kappa, sensitivity, specificity and confusion matrices were calculated using the ‘confusionMatrix’ function from the caret package (Kuhn 2008).

To illustrate shape differences, deformation vector plots were generated using the ‘plotRefToTarget’ function. These were used to visualise landmark displacement between the mean consensus shapes of the two species.

### 2.5 Image Analysis

An image analysis was conducted to examine variation in ventral coloration (VC) along the lateral edges of the disc. Ventral photographs of juvenile skate were selected based on image quality, resulting in a sample size of 29 (14 blue skate, 15 flapper skate). Using ImageJ (v1.54g; Schneider et al. 2012), area and mean greyscale measurements for the disc and VC region were recorded. Measurements were restricted to the right half of the disc and a single lateral VC band for each specimen, unless placement or image quality prevented accurate measurement (Figure 1D). The percentage area of VC was calculated relative to the total disc area as follows Equation (1):

(1) $$\text{VCArea}\left(\%\right)=\frac{\mathrm{Are}a_{\mathrm{VC}}}{\mathrm{Are}a_{\text{body}}}\times 100$$

VC darkness was defined as the mean greyscale value of the VC region. A scatterplot was used to visualise changes in VC size and darkness with length using the *ggplot2* package, and a Wilcoxin Rank Sum test was conducted to test species differences in VC area and darkness using the ‘wilcox. test’ function from the *stats* package (version 3.6.2, R Core Team, 2025).

## 3 Results

### 3.1 COI Barcoding

Molecular analyses using COI barcoding successfully identified 39 individuals as flapper skate and 46 as common blue skate ( *D. batis* ). Species assignments were supported by 12 diagnostic nucleotide positions within the COI fragment. Phylogenetic analysis recovered two well‐supported species‐specific clades with 100% bootstrap support, corroborating the molecular identification of all specimens (Figure S1). Notably, the two species never occurred within the same haul (Figure S2).

### 3.2 Morphometric Analysis

Following image sub‐setting, a total of 53 samples were retained for morphometric analysis, composed of 23 females and 30 males. Individuals ranged from 29.4 cm to 99.4 cm tail length, with an overall average length of 58.06 (± 14.95) cm recorded (Table S1).

#### 3.2.1 Full Disc

**FIGURE 2:**
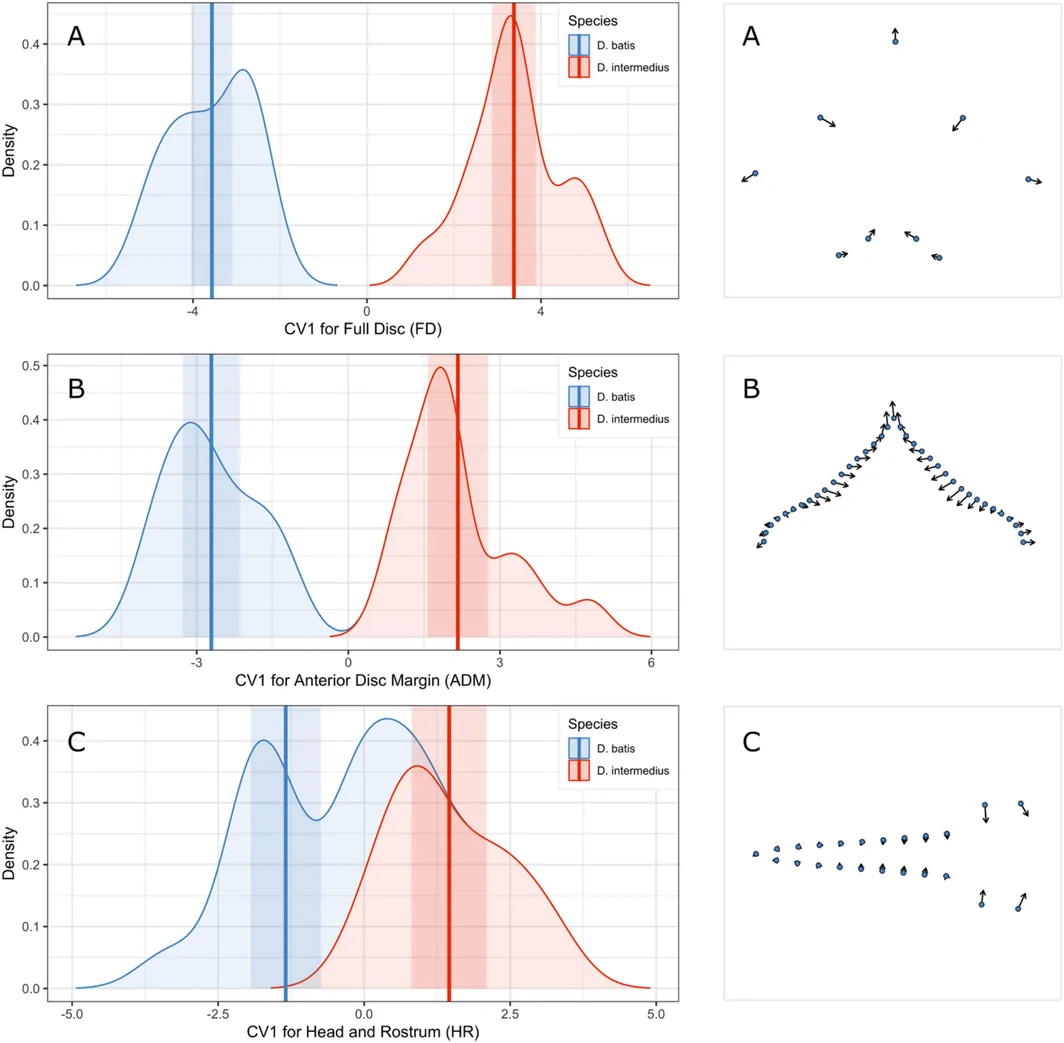
Morphological differentiation between juvenile common blue skate ( *Dipturus batis* ) and flapper skate ( *D. intermedius* ) across three morphological features: A. Full disc (FD), B. Anterior disc margin (ADM) and C. Head and rostrum (HR). Left: Kernel density distributions of CV1 scores for each species, with species mean CV1 scores indicated by vertical lines and 95% confidence intervals shown by shaded regions. Right: Deformation vector plots showing landmark displacement between species consensus configurations, with blue points representing the mean shape of *D. batis* .

Procrustes ANOVA indicated a significant effect of species on full disc shape (*F* = 7.86, *Z* = 4.58, *p* = 0.001, mean distance = 0.04), with species explaining 18.40% of the observed variation (R^2^ = 0.18). No significant effect of sex or centroid size was found (*p* > 0.05 in both cases; Table S3). Canonical variate analysis showed complete separation between *D. batis* and *D. intermedius* along the first canonical variate (CV1; Figure 2A). Leave‐one‐out cross‐validation correctly classified 100% of specimens (Sensitivity = 1, Specificity = 1, Kappa = 1), indicating high discriminatory performance. Differences in full body shape were characterised by a broader disc, greater anterior projection of the snout relative to the disc margin, increased concavity of the anterior disc margin, and a narrowing of the posterior disc apices in *D. intermedius* (Figure 2A).

#### 3.2.2 Anterior Disc Margin

Procrustes ANOVA indicated a significant effect of species on the shape of the anterior disc margin (*F* = 11.06, *Z* = 4.53, *p* = 0.01, mean distance = 0.04), with species explaining 31.00% of the observed variation (R^2^ = 0.31). No significant effect of sex or centroid size was found (*p* > 0.05 in both cases; Table S3). Canonical variate analysis showed minimal overlap between *D. batis* and *D. intermedius* along the first canonical variate (CV1; Figure 2B). Leave‐one‐out cross‐validation correctly classified 100% of specimens (Sensitivity = 1, Specificity = 1, Kappa = 1), indicating high discriminatory performance. Differences in anterior disc margin shape were characterised by a lengthening and narrowing of the head region, a more pronounced curvature towards the wing edge, and a lateral displacement of the disc corners in *D. intermedius* (Figure 2B).

#### 3.2.3 Head and Rostrum

Procrustes ANOVA indicated a significant effect of species on the shape of the head and rostrum (*F* = 5.08, *Z* = 3.08, *p* = 0.001, mean distance = 0.03), with species explaining 18.50% of the observed variation (R^2^ = 0.19). No significant effect of sex or centroid size was found (*p* > 0.05 in both cases; Table S3). However, substantial overlap between *D. batis* and *D. intermedius* was observed along the first canonical variate (CV1; Figure 2C). Leave‐one‐out cross‐validation correctly classified 76% of specimens (Sensitivity = 0.77 Specificity = 0.75; Kappa = 0.52), indicating moderate discriminatory performance based on head and rostrum shape. Differences in head and rostrum shape were characterised by a slight narrowing of the rostrum and reduced interorbital width in *D. intermedius* (Figure 2C).

Summaries of principal components and their contributions to the first canonical variate (CV1) for each trait are given in the appendices (Tables S2a–c). Ventral colouration measurements are given in Table S4.

### 3.3 Image Analysis

#### 3.3.1 Ventral Colouration

**FIGURE 3:**
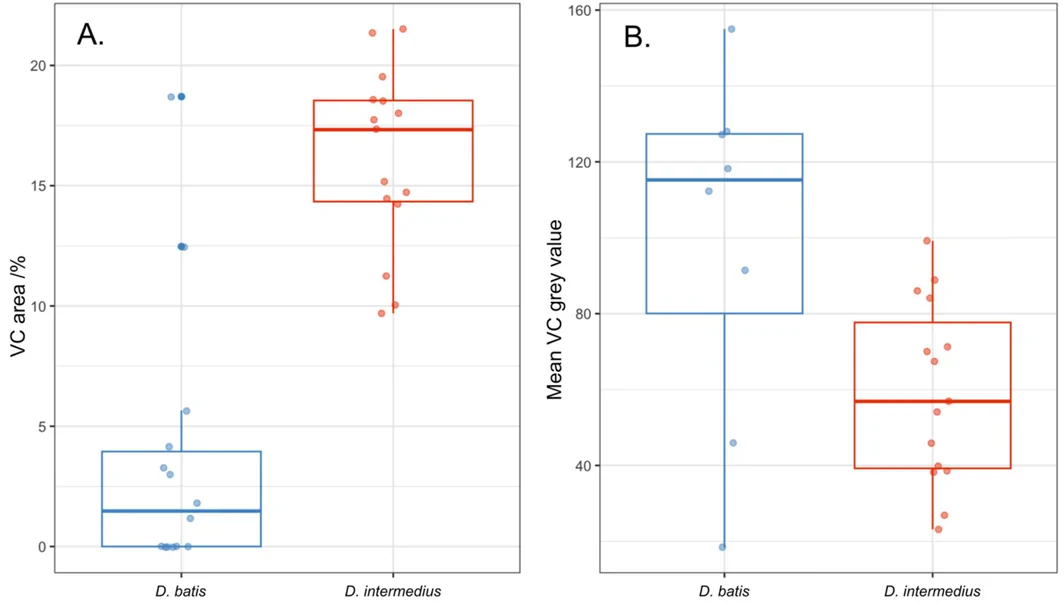
Comparison of ventral colouration (VC) area and mean greyscale value between juvenile common blue skate ( *Dipturus batis* ) and flapper skate ( *D. intermedius* ). Boxplots show species differences in A. percentage VC area and B. mean VC greyscale value, where lower values indicate darker pigmentation.

All juvenile flapper skate displayed dark bands along the postero‐lateral margins of the disc, covering 16.14% ± 3.76% of total body area with a mean greyscale value of 58.37 ± 23.82. In contrast, juvenile common blue skate exhibited limited ventral colouration, averaging 3.59% ± 5.54% coverage, and where present (*n* = 8), with lighter pigmentation (mean greyscale value = 99.57 ± 45.79; Table S4). Wilcoxon rank‐sum tests indicated these differences were significant in both cases (percentage VC area: *W* = 15, *p* = 8.74 × 10^−5^, mean VC greyscale value: *W* = 95, *p* = 0.02; Figure 3).

**FIGURE 4:**
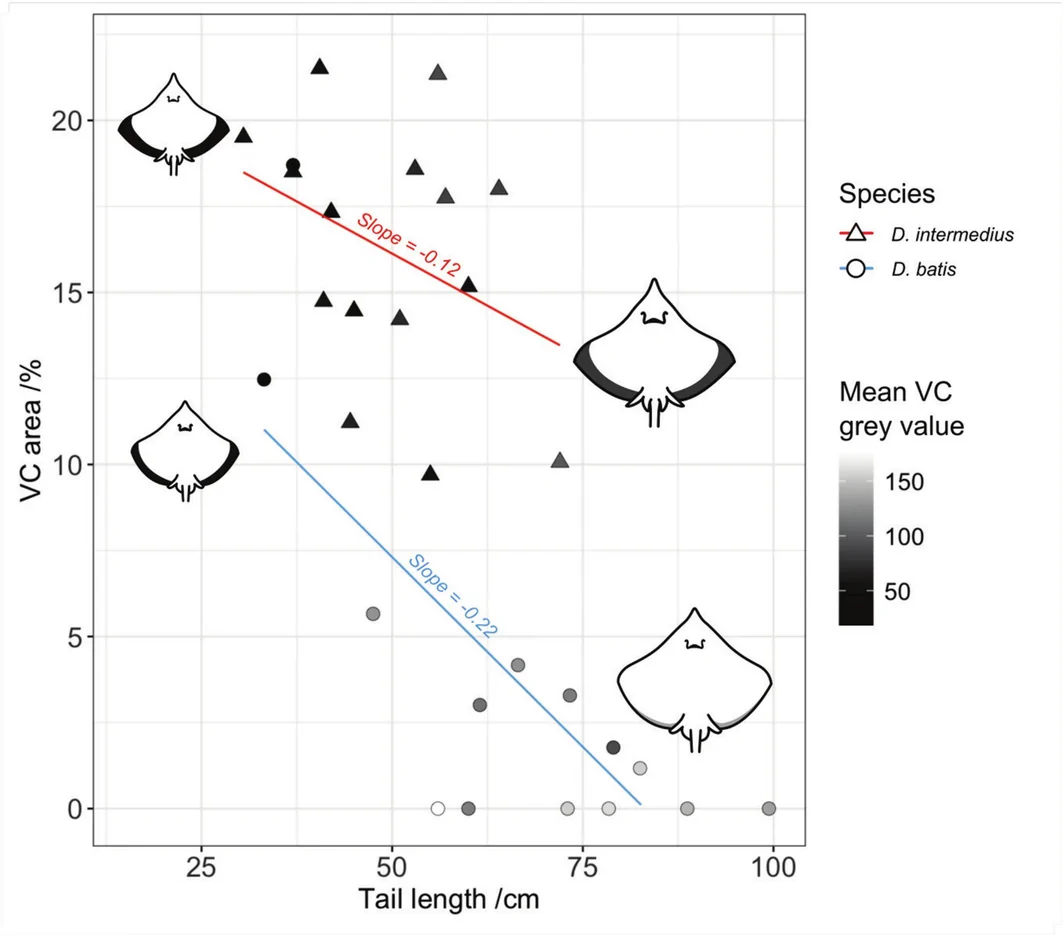
Relationship between ventral colouration (VC) area and tail length in common blue skate ( *Dipturus batis* ) and flapper skate ( *D. intermedius* ). Species‐specific regression lines for VC area are plotted with slope values. A linear model indicated a significant effect of length on VC area in the blue skate only (*p* = 0.002). Illustrations depict the reduction in VC area and changes in pigmentation intensity with increasing size for each species.

VC area decreased with total length in both species; however, the relationship was only significant in the common blue skate (*F* = 16.41, R^2^ = 0.58, *p* = 0.002, slope = −0.22), while the flapper skate exhibited a weaker, non‐significant trend (*F* = 1.92, R^2^ = 0.13, *p* = 0.19, slope = −0.12; Figure 4).

## 4 Discussion

This study aimed to develop a validated morphological framework for differentiating juvenile flapper and common blue skates by integrating morphological and molecular approaches. The analysis focused on identifying morphological traits that remain detectable in damaged specimens, ensuring the method's practical applicability in field conditions.

Based on our findings, four features are recommended for distinguishing juvenile blue and flapper skates: 1. Ventral coloration: A distinct dark lateral band is observed along the ventral disc margins of juvenile flapper skates, contrasting with a paler central region. In the common blue skate, this feature appears to occur only in the smallest individuals (< 40 cm TL) and is less pronounced or absent in those exceeding 50 cm total length, replaced instead by a uniformly pale ventral surface (Figure S3). 2. Disc shape: In the common blue skate, a compact, almost square‐shaped disc is observed; in the flapper skate, laterally elongated pectoral fins produce a rhombic disc that appears wider relative to its length. 3. Anterior disc margin: The anterior edge of the disc in juvenile flapper skate is strongly curved and in common blue skates this margin is comparatively straight. 4. Rostrum shape: Juvenile flapper skate tended to exhibit a more elongate, tapered rostrum than juvenile blue skate, although this feature showed considerable overlap between species.

In their morphological study, Iglésias et al. (2010) also noted species differences in rostrum shape and ventral banding, the latter feature was particularly evident in smaller individuals. Similarly, although not assessed in the present study, differences in dorsal colouration and wing blotches (ocelli) reported by Iglésias et al. (2010) appeared to be consistent diagnostic features for the juvenile (< 100 cm) individuals examined here. Although allometric shape variation and sexual dimorphism have been reported in batoids (Franklin et al. 2014; Gayford, Seamone, Irschick, et al. 2025; Gayford, Seamone, and Seidu 2025), neither log‐transformed centroid size nor sex significantly influenced the morphological features examined in this study. Nevertheless, our analyses were restricted to juvenile individuals, and future studies encompassing a broader size range could further evaluate ontogenetic changes in species‐specific morphology.

**FIGURE 5:**
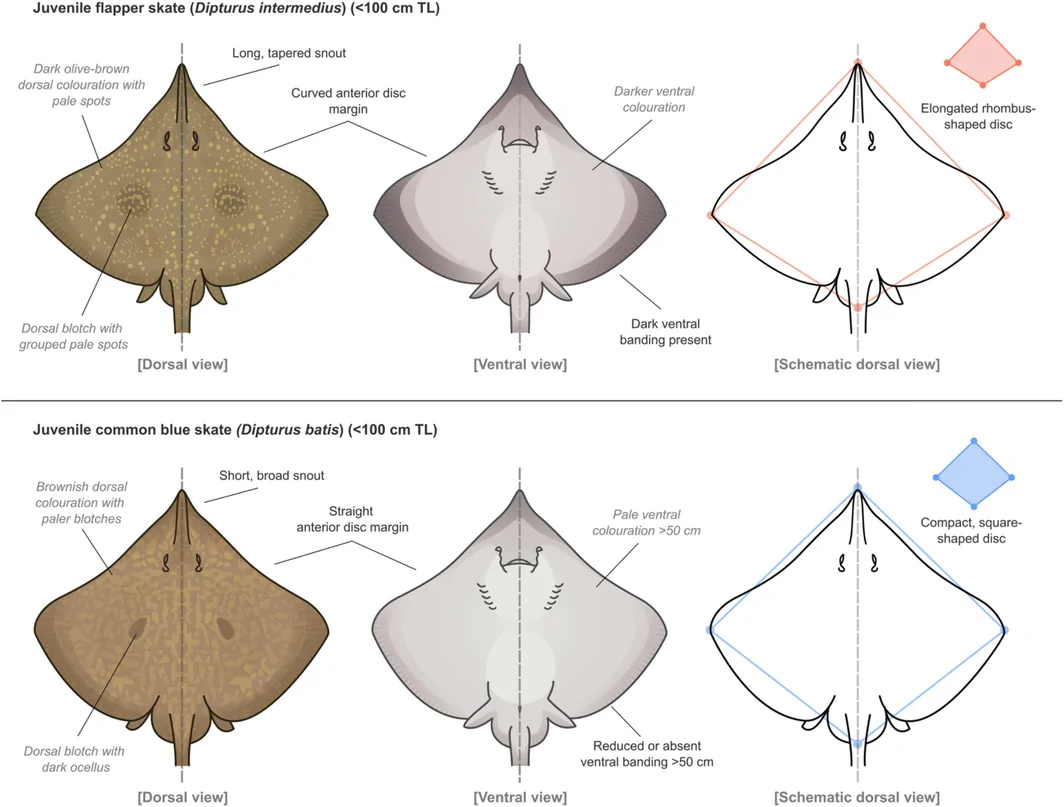
Diagnostic morphological features to distinguish juvenile flapper skate (*Dipturus intermedius*) and common blue skate ( *D. batis* ). Additional traits from Iglésias et al. (2010) are given in grey italics.

When combined with the identification criteria outlined by Iglésias et al. (2010), the traits identified here provide additional support for field‐based identification of members of the ‘common skate’ complex, particularly during juvenile life stages (Figure 5). These morphological characteristics are most appropriately interpreted as a suite of complementary features that, when considered together, improve species discrimination rather than as standalone diagnostic markers. Morphological traits for other superficially similar *Dipturus* species (e.g., the Norwegian skate, *D. nidarosiensis* and the long‐nosed skate, *D. oxyrinchus* ; see Iglésias et al. 2010) have not been formally evaluated within this framework. Given the overlapping distributions of these species in the NE Atlantic (Iglésias et al. 2010), future studies should assess whether the features presented here can contribute to accurate species identification across the genus.

### 4.1 Conservation Implications

Fisheries records indicate that substantial numbers of juvenile *Dipturus* skates are captured annually (e.g., see Frost et al. 2020; Ellis, Gordon, et al. 2024; Loca, Collins, et al. 2025), resulting in high rates of physical injury or mortality (Wilson et al. 2014). Given the strong link between juvenile recruitment and population dynamics in elasmobranchs (Ward‐Paige et al. 2012), such sustained fishing pressure likely poses a significant barrier to the recovery of these species. Effective spatial management strategies must target the critical habitats utilised by juveniles to protect these vulnerable life stages and facilitate species recovery (Martins et al. 2018; Dodd et al. 2022). Reliable species‐specific occurrence data is essential for informing these conservation measures, as the flapper and common blue skate, despite their morphological similarities, have distinct life history traits and ecological requirements that may lead them to utilise different critical habitats (Iglésias et al. 2010; Martins et al. 2018; Frost et al. 2020).

To date, widespread misidentification has affected data quality for juvenile flapper and common blue skate (Garbett et al. 2021; Ellis, McCully‐Philips, et al. 2024), resulting in knowledge gaps around their ecology and distribution. Although the discovery of a flapper skate egg nursery (Dodd et al. 2022) has provided some insight, little is known about the habitats these species rely on for their early life stages. By providing additional traits for reliable and accessible identification, the findings presented here enhance data accuracy for juvenile flapper and blue skates, support targeted conservation actions, and contribute to the effective management and recovery of these threatened species.

## Author Contributions

**Sophie Loca:** conceptualization (equal), data curation (equal), formal analysis (lead), investigation (equal), methodology (lead), writing – original draft (lead), writing – review and editing (lead). **Sara Fullerton:** conceptualization (equal), formal analysis (supporting), investigation (equal), writing – review and editing (supporting). **Caroline Bradley:** data curation (equal), methodology (supporting), writing – review and editing (supporting). **James Thorburn:** writing – original draft (supporting), writing – review and editing (supporting). **Amy Garbett:** writing – original draft (supporting), writing – review and editing (supporting). **Jack Burton:** data curation (equal). **Amber Keenan:** data curation (equal). **Daniel Wise:** data curation (supporting), writing – review and editing (supporting). **Lauren Smith:** data curation (supporting), writing – review and editing (supporting). **Chris Rickard:** data curation (supporting), writing – review and editing (supporting). **Thomas Barreau:** writing – review and editing (supporting). **Samuel Smith:** writing – review and editing (supporting). **Paulo A. Prodöhl:** data curation (equal), writing – review and editing (supporting). **Patrick C. Collins:** conceptualization (equal), investigation (equal), resources (lead), supervision (lead), writing – original draft (equal), writing – review and editing (supporting).

## Funding

This work was supported by EU INTERREG, IVA5060.

## Conflicts of Interest

The authors declare no conflicts of interest.

## Supporting information


**Figure S1:** Maximum‐likelihood phylogeny of the mitochondrial cytochrome *c* oxidase subunit I (COI) sequences generated from 85 juvenile *Dipturus* specimens collected during the 2021 Irish Groundfish Survey. The tree was reconstructed in Geneious Prime using the PhyML plugin under the Kimura two‐parameter (K80) substitution model with 1000 bootstrap replicates. Sequences formed two distinct clades corresponding to *Dipturus intermedius* and *D. batis* . The node separating the two species received 100% bootstrap support.
**Figure S2:** Map of IE‐IGFS 2021 haul locations where juvenile flapper (red dots) and common blue skate (blue dots) were recorded. Count represents number of individuals per haul.
**Figure S3:** Photographs of ventral colouration (VC) area in juvenile *Dipturus intermedius* (top) and *D. batis* (bottom). Measurements shown indicate the tail length measurements for each specimen.
**Table S1:** Samples used in morphological analyses of juvenile skate. ADM, anterior disc margin analysis, FD, full disc analysis, HR, head and rostrum analysis, TL, Tail length (cm), VC, ventral colouration analysis. Y and N indicate analyses where samples were included or excluded, respectively.
**Table S2a:** Summary of eigenvalues and percentage variance explained by principal components in the morphometric analyses of full disc (FD) shape in juvenile skate onboard the 2021 Irish Groundfish survey. Shaded rows indicate the PCs retained for the canonical variate analysis (CVA), with their relative contributions to the first canonical variate (CV1).
**Table S2b:** Summary of principal components in the morphometric analyses of anterior disc margin (ADM) shape in juvenile skate onboard the 2021 Irish Groundfish survey. Shaded rows indicate the PCs retained for the canonical variate analysis (CVA), with their relative contributions to the first canonical variate (CV1).
**Table S2c:** Summary of principal components in the morphometric analyses of head and rostrum (HR) shape in juvenile skate onboard the 2021 Irish Groundfish survey. Shaded rows indicate the PCs retained for the canonical variate analysis (CVA), with their relative contributions to the first canonical variate (CV1).
**Table S3:** Results of Procrustes ANOVA testing the effects of species, sex, and log‐transformed centroid size on juvenile skate shape variation, based on 1000 permutations. Analyses were conducted separately for A. full disc (FD) shape, B. anterior disc margin (ADM) shape and C. head and rostrum (HR) shape.
**Table S4:** Summary of ventral colouration (VC) measurements for juvenile *Dipturus batis* and *D. intermedius*.

## Data Availability

Datasets and R scripts supporting this study are available at: https://doi.org/10.5061/dryad.z08kprrtz. The COI sequences generated during this study have been deposited in GenBank under accession numbers PZ664735–PZ664819.
