# Supplementary material for: Spot the Difference: Morphometrics Reveals Key Traits for Distinguishing Two Cryptic Juvenile Skate
Source: Ecol Evol. 2026 Sep 4;16(9):e74305. doi: 10.1002/ece3.74305 (PMC13544996; doi:10.1002/ece3.74305)
Supplement: Supplementary file 1 [file ECE3-16-e74305-s001.docx]

**Appendix**

**Figures**

**Figure S1.** Maximum-likelihood phylogeny of the mitochondrial cytochrome *c* oxidase subunit I (COI) sequences generated from 85 juvenile *Dipturus* specimens collected during the 2021 Irish Groundfish Survey. The tree was reconstructed in Geneious Prime using the PhyML plugin under the Kimura two-parameter (K80) substitution model with 1,000 bootstrap replicates. Sequences formed two distinct clades corresponding to *Dipturus intermedius* and *D. batis*. The node separating the two species received 100% bootstrap support.

**Figure S2.** Map of IE-IGFS 2021 haul locations where juvenile flapper (red dots) and common blue skate (blue dots) were recorded. Count represents number of individuals per haul.

**Figure S3.** Photographs of ventral colouration (VC) area in juvenile *Dipturus intermedius* (top) and *D. batis* (bottom). Measurements shown indicate the tail length measurements for each specimen.

**Tables**

**Table S1.** Samples used in morphological analyses of juvenile skate. FD = full disc analysis, ADM = anterior disc margin analysis, HR = head and rostrum analysis, VC = ventral colouration analysis, TL = Tail length (cm). Y and N indicate analyses where samples were included or excluded, respectively.

| **ID** | **Species** | **Sex** | **TL** | **FD** | **ADM** | **HR** | **VC** |
| --- | --- | --- | --- | --- | --- | --- | --- |
| A5 | *D. intermedius* | M | 42.0 | Y | Y | N | Y |
| A6 | *D. intermedius* | M | 44.5 | Y | Y | Y | Y |
| A7 | *D. intermedius* | F | 37.0 | Y | Y | Y | Y |
| A8 | *D. intermedius* | M | 60.0 | N | N | N | Y |
| A9 | *D. intermedius* | M | 30.5 | N | N | N | Y |
| A10 | *D. intermedius* | F | 53.0 | N | Y | Y | Y |
| A11 | *D. intermedius* | F | 45.0 | Y | Y | Y | Y |
| A12 | *D. intermedius* | M | 55.0 | N | N | N | Y |
| A13 | *D. intermedius* | F | 51.0 | Y | N | N | Y |
| A14 | *D. intermedius* | M | 52.0 | Y | N | N | N |
| A15 | *D. intermedius* | F | 40.5 | N | N | N | Y |
| A18 | *D. intermedius* | F | 41.0 | Y | N | N | Y |
| A20 | *D. intermedius* | M | 47.0 | Y | Y | Y | N |
| A21 | *D. intermedius* | M | 44.0 | Y | Y | Y | N |
| J1 | *D. intermedius* | F | 56.0 | Y | Y | Y | Y |
| J2 | *D. intermedius* | M | 64.0 | Y | Y | N | N |
| J3 | *D. intermedius* | F | 61.0 | Y | N | N | N |
| J4 | *D. intermedius* | M | 60.0 | Y | N | N | N |
| J5 | *D. intermedius* | M | 69.0 | Y | Y | Y | N |
| J6 | *D. intermedius* | M | 64.0 | Y | N | N | Y |
| J7 | *D. intermedius* | M | 66.0 | N | Y | N | N |
| J10 | *D. intermedius* | F | 48.0 | Y | N | N | N |
| J20 | *D. intermedius* | M | 57.0 | Y | Y | Y | N |
| J21 | *D. intermedius* | M | 57.0 | N | Y | Y | Y |
| J22 | *D. intermedius* | M | 66.0 | N | N | Y | N |
| J23 | *D. intermedius* | M | 72.0 | Y | Y | N | Y |
| S1 | *D. batis* | F | 37.0 | N | Y | Y | Y |
| S5 | *D. batis* | F | 77.0 | Y | N | N | N |
| S6 | *D. batis* | F | 74.3 | Y | N | N | N |
| S7 | *D. batis* | F | 58.5 | N | N | Y | N |
| S9 | *D. batis* | F | 99.4 | Y | N | N | Y |
| S11 | *D. batis* | F | 47.5 | Y | Y | Y | Y |
| S15 | *D. batis* | F | 82.5 | Y | N | N | Y |
| S18 | *D. batis* | F | 78.4 | N | N | N | Y |
| S19 | *D. batis* | F | 74.5 | Y | N | N | N |
| S23 | *D. batis* | M | 56.0 | N | Y | Y | Y |
| S24 | *D. batis* | M | 60.0 | Y | N | N | Y |
| S25 | *D. batis* | F | 61.5 | Y | N | N | Y |
| S27 | *D. intermedius* | M | 38.1 | Y | Y | Y | N |
| S29 | *D. batis* | M | 73.0 | Y | Y | Y | Y |
| S30 | *D. batis* | F | 68.0 | Y | N | N | N |
| S31 | *D. batis* | M | 73.3 | N | N | N | Y |
| S33 | *D. batis* | F | 33.2 | Y | Y | Y | Y |
| S34 | *D. batis* | M | 53.3 | Y | Y | Y | N |
| S35 | *D. batis* | M | 58.2 | Y | Y | Y | N |
| S36 | *D. batis* | F | 62.2 | Y | N | N | N |
| S38 | *D. batis* | M | 56.9 | Y | Y | Y | N |
| S39 | *D. batis* | M | 29.4 | N | Y | Y | N |
| S41 | *D. batis* | M | 66.5 | Y | Y | Y | Y |
| S42 | *D. batis* | F | 88.7 | N | N | N | Y |
| S43 | *D. batis* | M | 58.3 | N | N | Y | N |
| S44 | *D. batis* | M | 79.0 | Y | N | N | Y |
| S45 | *D. batis* | M | 50.0 | Y | Y | Y | N |

**Table S2a.** Summary of eigenvalues and percentage variance explained by principal components in the morphometric analyses of full disc (FD) shape in juvenile skate onboard the 2021 Irish Groundfish survey. Shaded rows indicate the PCs retained for the canonical variate analysis (CVA), with their relative contributions to the first canonical variate (CV1).

| Component | Eigenvalue | Variance explained (%) | Cumulative variance (%) | Contribution to CV1 (%) |
| --- | --- | --- | --- | --- |
| PC1 | 6.61E-04 | 27.18 | 27.18 | 18.18 |
| PC2 | 4.98E-04 | 20.50 | 47.68 | 19.81 |
| PC3 | 3.53E-04 | 14.54 | 62.22 | 17.61 |
| PC4 | 2.19E-04 | 9.02 | 71.24 | 17.89 |
| PC5 | 1.95E-04 | 8.02 | 79.26 | 7.96 |
| PC6 | 1.39E-04 | 5.73 | 84.98 | 4.19 |
| PC7 | 1.05E-04 | 4.34 | 89.32 | 0.36 |
| PC8 | 7.37E-05 | 3.03 | 92.35 | 14.00 |
| PC9 | 6.41E-05 | 2.64 | 94.99 |  |
| PC10 | 3.94E-05 | 1.62 | 96.61 |  |
| PC11 | 2.98E-05 | 1.23 | 97.84 |  |
| PC12 | 2.64E-05 | 1.09 | 98.93 |  |
| PC13 | 1.71E-05 | 0.70 | 99.63 |  |
| PC14 | 9.01E-06 | 0.37 | 100.00 |  |

**Table S2b.** Summary of principal components in the morphometric analyses of anterior disc margin (ADM) shape in juvenile skate onboard the 2021 Irish Groundfish survey. Shaded rows indicate the PCs retained for the canonical variate analysis (CVA), with their relative contributions to the first canonical variate (CV1).

| Component | Eigenvalue | Variance explained (%) | Cumulative variance (%) | Contribution to CV1 (%) |
| --- | --- | --- | --- | --- |
| PC1 | 6.11E-04 | 37.44 | 37.44 | 52.56 |
| PC2 | 4.20E-04 | 25.75 | 63.19 | 7.39 |
| PC3 | 1.97E-04 | 12.05 | 75.24 | 19.16 |
| PC4 | 1.81E-04 | 11.06 | 86.31 | 5.22 |
| PC5 | 9.88E-05 | 6.05 | 92.36 | 15.67 |
| PC6 | 5.47E-05 | 3.35 | 95.71 |  |
| PC7 | 2.94E-05 | 1.8 | 97.51 |  |
| PC8 | 9.02E-06 | 0.55 | 98.06 |  |
| PC9 | 8.15E-06 | 0.5 | 98.56 |  |
| PC10 | 4.63E-06 | 0.28 | 98.85 |  |
| PC11 | 3.92E-06 | 0.24 | 99.09 |  |
| PC12 | 3.45E-06 | 0.21 | 99.3 |  |
| PC13 | 2.38E-06 | 0.15 | 99.44 |  |
| PC14 | 1.83E-06 | 0.11 | 99.56 |  |
| PC15 | 1.45E-06 | 0.09 | 99.65 |  |
| PC16 | 1.08E-06 | 0.07 | 99.71 |  |
| PC17 | 9.56E-07 | 0.06 | 99.77 |  |
| PC18 | 8.78E-07 | 0.05 | 99.82 |  |
| PC19 | 6.97E-07 | 0.04 | 99.87 |  |
| PC20 | 4.99E-07 | 0.03 | 99.9 |  |
| PC21 | 4.22E-07 | 0.03 | 99.92 |  |
| PC22 | 3.12E-07 | 0.02 | 99.94 |  |
| PC23 | 3.03E-07 | 0.02 | 99.96 |  |
| PC24 | 2.69E-07 | 0.02 | 99.98 |  |
| PC25 | 2.30E-07 | 0.01 | 99.99 |  |
| PC26 | 1.34E-07 | 0.01 | 100 |  |

**Table S2c.** Summary of principal components in the morphometric analyses of head and rostrum (HR) shape in juvenile skate onboard the 2021 Irish Groundfish survey. Shaded rows indicate the PCs retained for the canonical variate analysis (CVA), with their relative contributions to the first canonical variate (CV1).

| Component | Eigenvalue | Variance explained (%) | Cumulative variance (%) | Contribution to CV1 (%) |
| --- | --- | --- | --- | --- |
| PC1 | 5.62E-04 | 39.07 | 39.07 | 22.94 |
| PC2 | 2.71E-04 | 18.84 | 57.92 | 17.82 |
| PC3 | 1.63E-04 | 11.34 | 69.25 | 23.16 |
| PC4 | 1.48E-04 | 10.3 | 79.55 | 24.43 |
| PC5 | 7.89E-05 | 5.49 | 85.04 | 1.96 |
| PC6 | 7.26E-05 | 5.05 | 90.1 | 9.70 |
| PC7 | 3.93E-05 | 2.73 | 92.83 |  |
| PC8 | 2.71E-05 | 1.89 | 94.71 |  |
| PC9 | 2.05E-05 | 1.43 | 96.14 |  |
| PC10 | 1.49E-05 | 1.04 | 97.18 |  |
| PC11 | 9.82E-06 | 0.68 | 97.86 |  |
| PC12 | 7.37E-06 | 0.51 | 98.37 |  |
| PC13 | 4.73E-06 | 0.33 | 98.7 |  |
| PC14 | 3.57E-06 | 0.25 | 98.95 |  |
| PC15 | 2.99E-06 | 0.21 | 99.16 |  |
| PC16 | 2.59E-06 | 0.18 | 99.34 |  |
| PC17 | 2.22E-06 | 0.15 | 99.49 |  |
| PC18 | 1.75E-06 | 0.12 | 99.62 |  |
| PC19 | 1.57E-06 | 0.11 | 99.73 |  |
| PC20 | 1.38E-06 | 0.1 | 99.82 |  |
| PC21 | 9.32E-07 | 0.06 | 99.89 |  |
| PC22 | 6.85E-07 | 0.05 | 99.93 |  |
| PC23 | 5.83E-07 | 0.04 | 99.98 |  |
| PC24 | 3.53E-07 | 0.02 | 100 |  |

**Table S3.** Results of Procrustes ANOVA testing the effects of species, sex, and log-transformed centroid size on juvenile skate shape variation, based on 1,000 permutations. Analyses were conducted separately for **A.** full disc (FD) shape, **B.** anterior disc margin (ADM) shape, and **C.** head and rostrum (HR) shape.

| **Feature** | **Variable** | **Df** | **SumOfSqs** | **R2** | **F** | **Z** | **Pr(>F)** |
| --- | --- | --- | --- | --- | --- | --- | --- |
| **A. FD** | Species | 1 | 0.016 | 0.184 | 7.864 | 4.577 | 0.001 |
|  | Log(Centroid size) | 1 | 0.002 | 0.020 | 0.856 | -0.019 | 0.504 |
|  | Sex | 1 | 0.002 | 0.025 | 1.091 | 0.424 | 0.340 |
|  | Residual | 33 | 0.067 | 0.771 |  |  |  |
|  | Total | 36 | 0.087 | 1.000 |  |  |  |
|  |  |  |  |  |  |  |  |
| **B. ADM** | Species | 1 | 0.013 | 0.310 | 11.075 | 4.525 | 0.001 |
|  | Log(Centroid size) | 1 | 0.001 | 0.029 | 1.021 | 0.279 | 0.395 |
|  | Sex | 1 | 0.001 | 0.018 | 0.646 | -0.494 | 0.698 |
|  | Residual | 23 | 0.027 | 0.644 |  |  |  |
|  | Total | 26 | 0.042 | 1.000 |  |  |  |
|  |  |  |  |  |  |  |  |
| **C. HR** | Species | 1 | 0.006 | 0.185 | 5.082 | 3.079 | 0.001 |
|  | Log(Centroid size) | 1 | 0.001 | 0.031 | 0.863 | -0.077 | 0.532 |
|  | Sex | 1 | 0.001 | 0.020 | 0.555 | -0.762 | 0.774 |
|  | Residual | 21 | 0.026 | 0.764 |  |  |  |
|  | Total | 24 | 0.034 | 1.000 |  |  |  |

**Table S4.** Summary of ventral colouration (VC) measurements for juvenile *Dipturus batis* and *D. intermedius.*

| **ID** | **Body area (px)** | **VC area (px)** | **VC area (%)** | **Mean body greyscale value** | **Mean VC greyscale value** |
| --- | --- | --- | --- | --- | --- |
| A5 | 906058 | 157022 | 17.330 | 61.496 | 23.163 |
| A6 | 659097 | 73959 | 11.221 | 97.812 | 67.372 |
| A7 | 657530 | 121668 | 18.504 | 80.725 | 54.085 |
| A8 | 1393860 | 211497 | 15.173 | 82.069 | 45.817 |
| A9 | 847246 | 165326 | 19.513 | 72.840 | 38.175 |
| A10 | 632442 | 117508 | 18.580 | 112.203 | 70.070 |
| A11 | 771172 | 111558 | 14.466 | 71.802 | 26.850 |
| A12 | 1310577 | 127074 | 9.696 | 74.104 | 38.590 |
| A13 | 910278 | 129376 | 14.213 | 100.330 | 71.304 |
| A15 | 865126 | 186086 | 21.510 | 65.798 | 39.870 |
| A18 | 1124776 | 165816 | 14.742 | 102.133 | 56.889 |
| J1 | 1399641 | 298683 | 21.340 | 122.390 | 88.895 |
| J6 | 1265196 | 227694 | 17.997 | 121.043 | 84.062 |
| J21 | 1398594 | 248244 | 17.750 | 113.244 | 86.014 |
| J23 | 1412382 | 142185 | 10.067 | 118.511 | 99.175 |
| S1 | 1096173 | 205023 | 18.704 | 43.441 | 18.452 |
| S9 | 1593360 | 0 | 0 | 134.987 | - |
| S11 | 1649574 | 93366 | 5.660 | 179.379 | 128.024 |
| S15 | 1159389 | 13584 | 1.172 | 184.647 | 155.030 |
| S18 | 1388589 | 0 | 0 | 159.974 | - |
| S23 | 1521120 | 0 | 0 | 176.064 | - |
| S24 | 1869795 | 0 | 0 | 117.223 | - |
| S25 | 1598754 | 48126 | 3.010 | 113.334 | 112.239 |
| S29 | 1482891 | 0 | 0 | 154.397 | - |
| S31 | 1991463 | 65499 | 3.289 | 142.936 | 118.252 |
| S33 | 885936 | 110476 | 12.470 | 104.446 | 45.951 |
| S41 | 1528725 | 63741 | 4.170 | 135.091 | 127.153 |
| S42 | 1922259 | 0 | 0 | 144.612 | - |
| S44 | 1701042 | 30206 | 1.776 | 130.402 | 91.428 |
